# Associations between odontogenic and sinus pathologies – a low-dose CBCT study

**DOI:** 10.2340/aos.v84.43779

**Published:** 2025-06-03

**Authors:** Veli-Pekka Riekki, Mika T. Nevalainen, Marianne Haapea, Annina Sipola, Soili Kallio-Pulkkinen, Michaela K. Bode

**Affiliations:** a Department of Diagnostic Radiology, Oulu University Hospital, Oulu, Finland; b Research Unit of Health Sciences and Technology, Faculty of Medicine, University of Oulu, Oulu, Finland; c Medical Research Center Oulu, University of Oulu and Oulu University Hospital, Oulu, Finland; d Research Service Unit, Oulu University Hospital, Oulu, Finland; e Research Unit of Population Health, Faculty of Medicine, University of Oulu, Oulu, Finland; f Kallio & Pulkkinen Oy, Oulu, Finland

**Keywords:** Maxillary sinusitis, cone beam CT, odontogenic sinusitis, mucosal thickening, periodontal bone loss

## Abstract

**Objective:**

To evaluate maxillary sinus and odontogenic findings in low-dose CBCT, and to test if associations exist between these findings.

**Methods:**

From 263 consecutive CBCT scans, 212 were included. Evaluated odontogenic parameters were periapical lesions (PAI), marginal periodontal bone loss (PBL), root contact to the maxillary sinus, the presence of crowns, implants, defective restorations and extensive caries. Maxillary sinus findings were categorized as mucosal thickening (MT; generalized and localized), mucosal retention cysts, and opacification/fluid level. Crosstabulations and generalized estimating equations were used as statistical tests. Prevalence and bias adjusted kappas were calculated for inter- and intra-observer agreement.

**Results:**

The only dental finding statistically associated with sinus pathology when compared to healthy sinuses was root contact, which increased the risk for generalized MT (OR = 1.47, p = 0.015), mucosal retention cysts (OR = 2.60, p < 0.001) and opacification/fluid (OR = 1.76, p = 0.018). All parameters showed almost perfect or substantial intra- and interobserver reliability, except for PBL, where the former was moderate and the latter fair.

**Conclusion:**

Root contact was the only dental finding significantly associated with sinus pathology. Thus, there may be fewer associations between radiological dental findings and sinus pathology than previously thought. With the exception of PBL, all parameters demonstrated at least substantial intra- and interobserver reliability, indicating that the assessment is reliable overall.

## Introduction

Maxillary sinus inflammation has traditionally been associated with respiratory infections and allergies [1]. However, in the light of the existing literature, up to 40% of all chronic maxillary sinusitis and 45–75% of unilateral maxillary sinus opacification on computed tomography (CT) can be of dental origin [2], which is easily explained by the close anatomic proximity of premolar and molar teeth root apices to the maxillary sinuses. Many of the previous studies have associated maxillary sinus alterations to periapical lesions and/or periodontal bone loss (PBL) [3–15]. Contradicting or indefinite findings regarding the dental disease have also been reported, although to a lesser degree [16–18]. In a recent meta-analysis, the presence of periapical lesions increased the probability of sinus mucosal thickening and odontogenic maxillary sinusitis up to 2.4-fold and 1.7-fold, respectively [19].

The cone beam computed tomography (CBCT) is nowadays considered as the gold standard for identifying sinus problems of dental origin. The use of low-dose CBCT in the imaging of the paranasal sinuses can reduce the radiation dose received by the patients [20]. Many of the earlier CBCT studies have used standard imaging values. Low-dose CBCT in the evaluation of maxillary sinus pathology and dental findings was first reported in 2018 by Bornstein et al. [21]. Since then, this technique has become more common, with emerging evidence showing that low-dose CBCT can provide comparable diagnostic accuracy to standard CBCT [22, 23].

The purpose of this research was to study maxillary sinus pathology and dental findings with low-dose CBCT in a series of consecutive patients in a retrospective setting, and, most importantly, to evaluate the associations between them. In addition, attention was paid on the reliability of the evaluation.

## Materials and methods

### Study population and image analysis

Consecutive sinus CBCT scans from a single centre (using a Planmeca Promax 3D Max unit with a low dose protocol) available between January and July 2019 were assessed retrospectively (*n* = 263). A mean tube current of 2.0 mA (range 2 – 3 mA) and a voltage of 120 kV were utilised. The average exposure time was 3.95 s (range 3.92 – 8.05 s). Other parameters included slice thickness and isotropic voxel size of 0.4 mm and 324 × 324 matrix. The exclusion criteria were as follows: patients lacking premolar and molar teeth (*n* = 28), trauma (*n* = 7), tumours (*n* = 4), age under 15 years (*n* = 4), scans taken for dacryocystorhinostomy (*n* = 4), and scans of poor quality due to artifacts (*n* = 4). Therefore, the CBCT scans of 212 patients, 87 (41.0%) males and 125 (59.0%) females, age range between 15 and 80 years (mean 42.4, standard deviation 16.0) were included in this study.

All the CBCT images were analysed by the same general radiologist (VPR), who also re-analysed 20 scans for intra-observer reliability. The same 20 scans were also analysed by oral and maxillofacial radiologist (SK-P) in order to determine inter-observer reliability. Prior analysis, a brief meeting was held between raters in order to familiarise the general radiologist with the evaluation of dental CBCT. Calibration was done by printed instructions and reference images illustrating examples of the possible morphological changes. Analysis was carried out with dedicated radiological workstation and software (neaView Radiology, Neagen Ltd., Helsinki, Finland) by using multiplanar reformation (MPR).

The study was conducted following the ethical principles stated in the Declaration of Helsinki. An institutional data permit was obtained. Non-interventional studies based on register data do not need approval from Ethics Committee in Finland. Informed consent was waived, since the integrity of a person was not violated and patients were not contacted.

### Evaluation of sinuses

The maxillary sinus mucosal thickness (MT) for each tooth was measured. An MT of 1 mm or less was considered normal. Other categories were: MT 1.1–2 mm; MT 2.1–4 mm; MT 4.1–10 mm; and MT over 10 mm. MT was considered localised when it involved one to two adjacent teeth, while generalised MT involved the whole sinus floor. Mucosal retention cysts were determined as dome-shaped hypodensities. Complete opacification and the fluid level were analysed and grouped together due to small numbers.

### Evaluation of teeth

In total, 1,565 teeth including eight implants (779 premolars and 786 molars) were evaluated.

**Figure 1:**
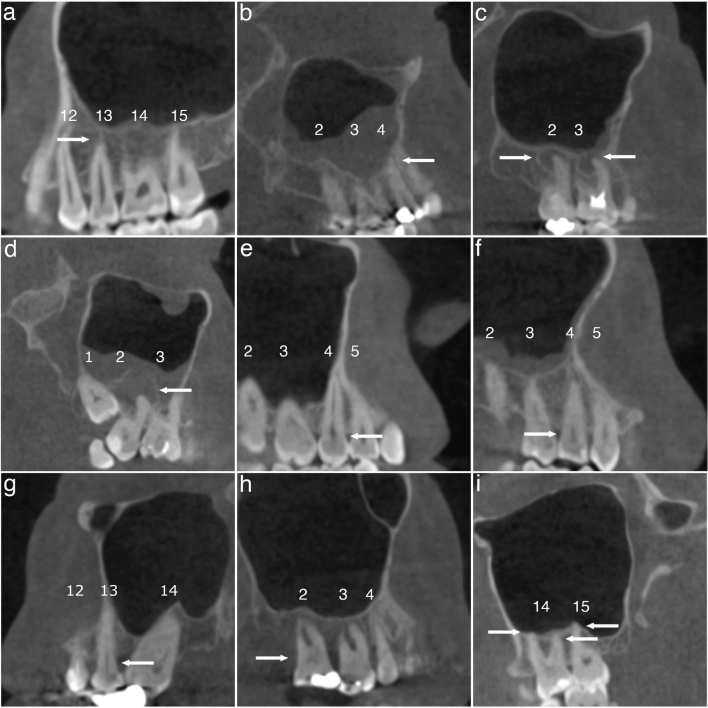
Representative CBCT scans of dental findings. Categories of periapical lesions: (a) normal; (b) widening of the radiolucent periodontal ligament space and minor changes of radiopaque lamina dura; (c) periodontitis with well-defined and corticated radiolucent area; (d) severe periodontitis with exacerbating structures. Categories of periodontal bone loss; (e) normal (2 mm or less); (f) mild (2.1 – 3 mm); (g) moderate (3.1 – 5 mm); (h) severe (more than 5 mm). (i) At least one of the tooth roots in contact with the maxillary sinus floor. Teeth are numbered according to World Dental Federation notation (FDI) and each category/finding is indicated by arrows.

Periapical lesions were classified in four categories (modified from Orstavik et al. [24]): 0 = normal; 1 = widening of the radiolucent periodontal ligament space and minor changes of radiopaque lamina dura; 2 = periodontitis with well-defined and corticated radiolucent area; and 3 = severe periodontitis with exacerbating structures (i.e. large diffuse area of bone destruction in the apical surrounding bone) (Figure 1a–d). Later, categories 0–1 and 2–3 were combined.

*Marginal PBL* was categorised as follows (modified from Goller-Bulut et al. [7]): 0 = normal (2 mm or less); 1 = mild (2.1 – 3 mm); 2 = moderate (3.1 – 5 mm); and 3 = severe (more than 5 mm). Later, classes 0–1 and 2–3 were combined (Figure 1e–h)

*The relationship of teeth with the maxillary sinus* was recorded as no contact or at least one of the tooth roots in contact with the maxillary sinus floor (Figure 1i).

The presence of *crowns, implants, defective restorations, root canal treatment*, and *extensive caries* were reported. Root canal therapy was found to be inadequate, if there were untreated root canals (both major and accessory) or if the root filling remained at least 2 mm from the radiographic tip or extended beyond the tip or if the density of the filling was insufficient (sparse). Due to the small number of implants (*n* = 8), they were excluded from the significance testing.

### Statistical analyses

Crosstabulations were used to present the distributions of laterality, sex, age group, and odontogenic findings in relation to maxillary sinus pathology. Generalised estimating equations (GEE) with a binary logistic link function (patient as a subject variable, teeth as a within factor variable, and each maxillary sinus pathology separately as a dependent variable) were used to evaluate the relationship of sex, age, and odontogenic findings with maxillary sinus pathology further. When evaluating effect of odontogenic findings, each sinus pathology was compared separately to normal sinuses. The effect of age and gender to sinus findings was also calculated. A *p*-value of less than 0.05 was considered to be statistically significant. Commercial software (SPSS, version 29.0, SPSS Inc., Chicago, IL, USA) was used to conduct the analyses.

Prevalence and bias adjusted kappas (PABAK) were calculated for inter-observer and intra-observer agreement using R version 4.3.0 (https://cran.r-project.org) accompanied with the ‘EpiR’ package. The PABAK values were interpreted as follows: 0.01–0.2 slight agreement; 0.21–0.40 fair agreement; 0.41–0.60 moderate agreement; 0.61–0.80 substantial agreement; and 0.81–1.00 almost perfect agreement.

## Results

### Sinus pathology

Of the 424 maxillary sinuses in the study, 356 (84.4%) showed some abnormalities. Detailed data on the distribution of the sinus findings are presented in Table 1. Maxillary sinus pathology was seen bilaterally in 161 (75.9%) and unilaterally in 34 (16.0%) patients. Generalised MT was the most frequent finding, occurring in 65.6% of the patients. Of these, 45.3% (63/139) occurred bilaterally. Only 2.3% (*n* = 2) of the men and 12.0% (*n* = 15) of the women had no sinus findings. Men presented with more retention cysts (Odds Ratio [OR] = 2.2, *p* = 0.008), while other sinus pathology did not show statistically significant differences between genders. Age did not relate to any of the sinus alterations.

**Table 1:** Distributions of laterality, sex, and age within the sinus findings in patient specific data (*n* = 212).

	Sinus findings *N* (%)					
	Healthy	Localised mucosal thickening	Generalised mucosal thickening	Cyst	Fluid level or diffuse opacification	Any sinus finding
Total	17 (8.0)	43 (20.3)	139 (65.6)	92 (43.4)	48 (22.6)	195 (92.0)
Laterality						
- Unilateral	n.a.	38 (17.9)	76 (35.8)	72 (34.0)	30 (14.2)	34 (16.0)
- Bilateral	n.a.	5 (2.4)	63 (29.7)	20 (9.4)	18 (8.5)	161 (75.9)
Sex						
- Female	15 (12.0)	26 (20.8)	79 (63.2)	49 (39.2)	28 (22.4)	110 (88.0)
- Male	2 (2.3)	17 (19.5)	60 (69.0)	43 (49.4)	20 (23.0)	85 (97.7)
OR (95% CI)^1^	1.0	1.21 (0.65–2.25)	1.55 (0.91–2.63)	2.18 (1.22–3.89)	1.56 (0.75–3.37)	1.62 (1.02–2.60)
Age, mean (SD)	45.7 (15.3)	41.9 (17.2)	42.2 (15.5)	41.0 (15.4)	39.8 (16.7)	42.1 (16.1)
OR (95% CI)^2^	1.0	0.91 (0.75–1.09)	1.02 (0.87–1.21)	0.87 (0.73–1.03)	0.84 (0.66–1.06)	0.95 (0.82–1.09)
Age group, years						
15–25	1 (3.4)	8 (27.6)	18 (62.1)	11 (37.9)	9 (31.0)	28 (96.6)
26–40	6 (7.4)	16 (19.8)	53 (65.4)	39 (48.1)	20 (24.7)	75 (92.6)
41–60	6 (9.4)	11 (17.2)	44 (68.8)	28 (43.8)	10 (15.6)	58 (90.6)
> 60	4 (10.5)	8 (21.1)	24 (63.2)	14 (36.8)	9 (23.7)	34 (89.5)

OR (95%) CI = odds ratio (95% confidence interval) from the generalised estimating equations.

OR: odds ratio; CI: confidence interval; SD: standard deviation.

1 Female as a reference group.

2 OR for 10 years increase.

### The relationship of sinus pathology with odontogenic findings

Among 1,565 examined teeth (including eight implants), 73.8% (*n* = 1,155) presented with maxillary sinus pathology. The distribution of odontogenic and sinus findings, and their relationship to each other is presented in Table 2. The most common sinus pathology was generalised MT. The only dental finding associated statistically significantly with sinus pathology when compared to healthy sinuses was root contact to the maxillary sinus floor. The root contact increased the risk of generalised MT (odds ratio [OR] = 1.47, *p* = 0.015), mucosal retention cysts (OR = 2.60, *p* < 0.001), and fluid/opacification (OR 1.76, *p* = 0.018) (Table 3).

**Table 2:** Distribution of odontogenic and sinus findings and their relationship to each other.

Odontogenic findings	Sinus pathology					Total
	Normal	Localised mucosal thickening	Generalised mucosal thickening	Retention cyst	Fluid or opacification	
	*N* (%)	*N* (%)	*N* (%)	*N* (%)	*N* (%)	*N* (%)
Total*						
Right	196 (24.9)	48 (6.1)	308 (39.1)	120 (15.3)	116 (13.7)	788 (100.0)
Left	214 (27.5)	27 (3.5)	297 (38.2)	125 (16.1)	114 (14.7)	777 (100.0)
**Periapical lesion**						
Right						
0–1	184 (25.1)	43 (5.9)	289 (39.4)	111 (15.1)	106 (14.5)	733 (100.0)
2–3	11 (22.4)	3 (6.1)	16 (32.7)	9 (18.4)	10 (20.4)	49 (100.0)
Left						
0–1	207 (27.5)	27 (3.6)	289 (38.4)	120 (15.9)	110 (14.6)	753 (100.0)
2–3	5 (22.7)	0 (0.0)	8 (36.4)	5 (22.7)	4 (18.2)	22 (100.0)
**Marginal bone loss**						
Right						
0–1	72 (25.7)	19 (6.8)	92 (32.9)	45 (16.1)	52 (18.6)	280 (100.0)
2–3	123 (24.5)	27 (5.4)	213 (42.4)	75 (14.9)	64 (12.7)	502 (100.0)
Left						
0–1	69 (28.0)	8 (3.3)	83 (33.7)	47 (19.1)	39 (15.9)	246 (100.0)
2–3	143 (27.0)	19 (3.6)	214 (40.5)	78 (14.7)	75 (14.2)	529 (100.0)
**Root contact**						
Right						
No	73 (34.0)	11 (5.1)	81 (37.7)	21 (9.8)	29 (13.5)	215 (100.0)
Yes	122 (21.5)	35 (6.2)	224 (39.5)	99 (17.5)	87 (15.3)	567 (100.0)
Left						
No	72 (34.3)	6 (2.9)	84 (40.0)	22 (10.5)	26 (12.3)	210 (100.0)
Yes	140 (24.8)	21 (3.7)	213 (37.7)	103 (18.2)	88 (15.6)	565 (100.0)
**Root canal therapy**						
Right						
No	185 (25.1)	45 (6.1)	287 (38.9)	112 (15.2)	109 (14.8)	738 (100.0)
Yes	11 (22.4)	3 (6.1)	21 (42.9)	7 (14.3)	7 (14.3)	49 (100.0)
Left						
No	203 (27.3)	27 (3.6)	284 (38.2)	120 (16.2)	109 (14.7)	743 (100.0)
Yes	11 (32.4)	0 (0.0)	13 (38.2)	5 (14.7)	5 (14.7)	34 (100.0)
**Restoration**						
Right						
No	185 (25.3)	43 (5.9)	282 (38.6)	111 (15.2)	109 (14.9)	730 (100.0)
Yes	10 (19.2)	3 (5.8)	23 (44.2)	9 (17.3)	7 (13.5)	52 (100.0)
Left						
No	200 (27.1)	27 (3.7)	281 (38.1)	119 (16.1)	110 (14.9)	737 (100.0)
Yes	12 (31.6)	0 (0.0)	16 (42.1)	6 (15.8)	4 (10.5)	38 (100.0)
**Crowns**						
Right						
No	177 (24.9)	43 (6.0)	275 (38.6)	114 (16.0)	103 (14.5)	712 (100.0)
Yes	18 (25.7)	3 (4.3)	30 (42.9)	6 (8.6)	13 (18.6)	70 (100.0)
Left						
No	186 (26.6)	23 (3.3)	266 (38.0)	116 (16.6)	109 (15.6)	700 (100.0)
Yes	26 (34.7)	4 (5.3)	31 (41.3)	9 (12.0)	5 (6.7)	75 (100.0)
**Extensive caries**						
Right						
No	192 (24.9)	46 (6.0)	304 (39.4)	115 (14.9)	114 (14.8)	771 (100.0)
Yes	3 (27.3)	0 (0.0)	1 (9.1)	5 (45.5)	2 (18.2)	11 (100.0)
Left						
No	209 (27.3)	27 (3.5)	292 (38.2)	124 (16.2)	113 (14.8)	765 (100.0)
Yes	3 (30.0)	0 (0.0)	5 (50.0)	1 (10.0)	1 (10.0)	10 (100.0)
**Implants**						
Right						
No	195 (24.9)	46 (5.9)	305 (39.0)	120 (15.3)	116 (14.8)	782 (100.0)
Yes	1 (16.7)	2 (33.3)	3 (50.0)	0 (0.0)	0 (0.0)	6 (100.0)
Left						
No	212 (27.4)	27 (3.5)	297 (38.3)	125 (16.1)	114 (14.7)	775 (100.0)
Yes	2 (100.0)	0 (0.0)	0 (0.0)	0 (0.0)	0 (0.0)	2 (100.0)

* Total number of teeth used is 1,565, including eight implants. The implants have been excluded from odontogenic findings categories other than implants, that is, *n* = 1,557.

**Table 3:** Associations of odontogenic findings with sinus findings when compared to healthy sinuses.

Odontogenic findings	Localised mucosal thickening		Generalised mucosal thickening		Retention cyst		Fluid or opacification	
	OR (95% CI)	*P*	OR (95% CI)	*P*	OR (95% CI)	*P*	OR (95% CI)	*P*
Periapical lesion	1.05 (0.35–3.16)	0.935	1.01 (0.49–2.08)	0.968	1.48 (0.68–3.23)	0.323	1.58 (0.70–3.57)	0.268
Marginal bone loss	0.90 (0.53–1.54)	0.707	1.29 (0.86–1.95)	0.222	0.88 (0.56–1.40)	0.590	0.81 (0.45–1.45)	0.479
Contact	1.82 (0.94–3.54)	0.077	1.47 (1.08–2.00)	0.015*	2.60 (1.58–4.28)	<0.001*	1.76 (1.10–2.81)	0.018*
Root canal therapy	0.75 (0.23–2.43)	0.631	1.05 (0.57–1.92)	0.881	0.90 (0.43–1.90)	0.784	0.96 (0.44–2.11)	0.925
Restoration	0.75 (0.21–2.63)	0.653	1.21 (0.63–2.33)	0.563	1.14 (0.56–2.31)	0.714	0.88 (0.38–2.01)	0.761
Crowns	0.88 (0.32–2.37)	0.793	0.93 (0.49–1.78)	0.827	0.54 (0.27–1.07)	0.078	0.70 (0.26–1.89)	0.481
Extensive Caries	n.a.	n.a.	0.67 (0.20–2.31)	0.529	1.68 (0.49–5.79)	0.413	0.88 (0.27–2.90)	0.838

OR: odds ratio; CI: confidence interval.

* *p* < 0.05, *p*-values and estimates from generalised estimating equations.

### Periapical lesions

Due to the small number of severe grade 3 periapical lesions (*n* = 9), they were combined with moderate grade 2 lesions (*n* = 62). The severity of periapical lesion did not increase the likelihood of maxillary sinus pathology (Table 3). Root canal therapy and defective restorations were found in 37 (52.1%) and 33 (46.5%) of gr 2–3 periapical lesions, respectively. The root canal therapy was inadequate in 25 (30.1%) of all teeth with root canal therapy. In all, 17 (68.0%) out 25 teeth with inadequate root canal therapy and 20 (34.5%) out of 58 teeth with adequate root canal therapy had grade 2–3 periapical lesion (OR 4.04, *p* = 0.006). Overall, there were few teeth with root canal therapy and other restorations, and the numbers decreased even more when grouped according to the severity of periapical lesions. Periapical lesion on its own or combined with root canal therapy or defective restorations was not statistically associated with the presence of any sinus pathology (results not shown). In addition, inadequate root canal therapy did not associate with sinus findings.

### Marginal bone loss (PBL)

PBL gr 0 was seen in 64 (4.1%), PBL gr 1 in 462 (29.7%), PBL gr 2 in 855 (54.9%), and PBL gr 3 in 176 (11.3%) of the examined teeth (*n* = 1,557), respectively. To reach clinically reasonable conclusions, the cases with no or minor PBL and those cases with moderate or severe bone loss were grouped. The proportion of PBL gr 2–3 was the highest within generalised mucosal thickening (right side 42.4% and left side 40.5%; Table 2). However, there was no statistically significant association with PBL and sinus pathology when compared to healthy sinuses (Table 3).

### Intra- and inter-observer agreement

Intra-observer agreement was almost perfect for localised MT, retention cysts, maxillary sinus fluid/opacification, periapical lesion, contact of tooth root/roots to the maxillary sinus floor, defective restoration and extensive caries; it was substantial for generalised diffuse MT and, finally, moderate for marginal bone loss. Inter-observer agreement was only fair for marginal bone loss, substantial for generalised MT and defective restoration, and almost perfect for other parameters. The numerical data are summarised in Table 4.

**Table 4:** Prevalence and bias adjusted kappa (PABAK) values for inter-observer and Intra-observer agreement for sinus and odontogenic findings.

	PABAK (95% confidence interval)	
	Intra-observer	Inter-observer
Local MT	0.893 (0.795–0.953)	0.812 (0.695–0.895)
Diffuse MT	0.653 (0.513–0.767)	0.624 (0.480–0.743)
Cyst	0.813 (0.697–0.896)	0.826 (0.711–0.905)
Fluid or opacification	0.987 (0.927–1.000)	0.852 (0.743–0.925)
Periapical lesion	0.973 (0.905–0.997)	0.973 (0.905–0.997)
Marginal bone loss	0.493 (0.339–0.628)	0.342 (0.177–0.493)
Contact	0.905 (0.809–0.961)	0.808 (0.689–0.893)
Defective restoration	0.907 (0.812–0.962)	0.699 (0.562–0.806)
Extensive caries	0.880 (0.778–0.944)	0.863 (0.755–0.933)

MT: mucosal thickness; PABAK: prevalence and bias adjusted kappas.

## Discussion

In this study, we aimed to evaluate maxillary sinus and odontogenic findings in low-dose CBCT, and to test if associations exist between these findings. The main finding of our study – somewhat surprisingly – was that only root contact to the maxillary sinus floor was statistically associated with sinus pathology when compared to healthy sinuses. Reliability for marginal bone loss was moderate for intra-observer and fair for inter-observer, while for other parameters the reliability was almost perfect or at least substantial.

Mucosal thickening was the most common finding with 76% at patient level and 59% at sinus level. The percentages are almost identical compared to recent article by Betin-Noriega et al. [5] (71 and 53%, respectively), however, lower prevalence rates have also been reported [3, 8, 11, 14]. There is still no full consensus on what constitutes pathological maxillary sinus mucosal thickening on CBCT. In addition, mucosal thickening can also be present without any symptoms. Some studies, the current study included, have used a threshold of 1 mm [7, 9, 10], while others have considered the upper limit of normal mucosal thickening to be 2 mm [5, 11, 12, 18] or even 3 mm [4, 25–27]. There is also great variability in study designs in other respects, for example the degrees or classification of sinus mucosal thickening or other diagnostic criteria of sinus disorder, as laudably presented in a systematic review and meta-analysis by Penarrocha-Oltra et al. [19]. In the current study, we wanted to acknowledge the presence of both localised and generalised MT similarly to Nascimento et al. [9], who associated the former to PBL and the latter to periapical lesions. In our study, no such associations were found. Only root contact to the maxillary sinus floor was associated with sinus pathology when compared to healthy sinuses. Previously, the proximity of roots with endodontic infection has correlated positively to sinus pathology [6, 9, 16, 28], although contradicting results have also been reported [8, 26].

The only demographic association in our study concerning sinus findings was between male gender and cysts. In previous studies, the association of cysts with odontogenic findings and demographics has been variable [9, 10, 26, 27, 29]. Some of the previous studies have found age [8, 30], male gender [9, 26, 31] or both [3, 11] to be associated with mucosal sinus thickening.

In a recent study by Wu et al. [32], the most common oral aetiologies of odontogenic sinusitis were endo-periodontal lesions (49.5%), apical periodontitis (AP) (32.0%), and periodontitis (8.7%). AP is often a chronic asymptomatic disease. The global prevalence of AP in the adult population was 52% at the individual level and 5% tooth level in a recent systematic review and meta-analysis [33]. The prevalence was higher in root filled teeth, being 39%, whereas only 3% of non-treated teeth had periapical lesions [33]. In our study, root canal therapy or restorations did not associate with sinus or dental pathology. Periodontal disease has previously been associated with sinus MT in many [4, 5, 7, 9–11], but not all [16–18] articles. Periapical lesions have on many occasions been associated with sinus MT [3, 5–8, 13, 14], however, some studies have also failed to show this association undeniably or at all [4, 10, 26]. Not all dental lesions and sinus mucosal alterations are clinically significant, and the most important question is which dental findings correlate with symptomatic sinusitis. Many of the existing studies reporting relationship between maxillary sinusitis and dental status have only included the radiological findings [5, 18], which is also the weakness of our study.

What explains the weak association of sinus and dental findings in the study? In Finland, low-cost public dental care covers the whole population and everyone under the age of 18 receives it free of charge. Globally, this is exceptional, and many previous studies come from countries where dental care is not so well organised and available for all. Thus, our study population may have healthier teeth when compared to the rest of the world. It may also be that our patients are referred to CBCT with fewer symptoms or clinical findings than is customary.

In a study by Bornstein et al. [21], low-dose protocol was shown to be feasible in analysing dentition in the posterior maxilla and changes in maxillary sinuses, with intra-observer reliability being almost perfect. In our study, intra- and inter-observer variability were acceptable, except for marginal bone loss. This might be due to the anatomically challenging area with great variability, where measured parameters are not necessarily unequivocally definable and are prone to more subjective interpretation. In addition, it is reasonable to expect that an oral and maxillofacial radiologist may be more skilled in evaluating dentition compared to a general radiologist.

There are some limitations in this study. Due to retrospective study design, we lacked clinical correlation of our imaging findings. Moreover, the patient material can be biased, since CBCT studies are nowadays ordered on rather loose grounds. The low amount of sinusitis or more severe odontogenic findings hampered the statistical analysis. The fact that one of the readers was a general radiologist and the other an oral and maxillofacial radiologist might partly explain the low reliability of the PBL analysis. In addition, the number of cases used for inter- and intra-observer variability calculations was quite low.

To conclude, the association of radiological dental findings with sinus pathology may not always be so evident, and the prevalence of odontogenic sinus findings is not necessarily so common in all populations. In addition, diagnosis and grading of marginal bone loss was not consistent. Therefore, a thorough clinical evaluation is essential, as interpreting and understanding the significance of imaging findings can be difficult.

## Data Availability

The data that support the findings of this study are not openly available due to reasons of sensitivity. However, they are available from the corresponding author upon reasonable request. Data are stored in controlled access data storage at Oulu University Hospital.
